# The humanity of science

**DOI:** 10.7554/eLife.27982

**Published:** 2017-05-04

**Authors:** Indira M Raman

**Affiliations:** Department of Neurobiology, Northwestern University, Evanston, United States

**Keywords:** Living science, humanities, diversity, careers in science

## Abstract

Science can provide cures and improve health, and it can also make us more humane.

Several years ago, a scientific colleague remarked upon his experience reviewing the portfolios of humanities professors. “Some of them are studying books that are, like, two hundred years old!” he exclaimed. His incredulous synopsis of the humanist endeavor reflected a pride in science’s inherently progressive mission, which literally presses into the future rather than analyzing the past. Indeed, many scientists often display a kind of glee at the prospect of overturning ideas historically thought to be true. My impression is that it is this urge to drive forward, with the apparent willingness to discard whatever came before, that gives science its own caricatured image of being cold or even inhumane. As such, scientific exploration is often viewed with suspicion by non-scientists, even those who desire the benefits of technology. To me, however, science has stronger links to humanist ideals, which go beyond providing comforts and conveniences. Humanists study how we live and what makes us humane, as distinct (or not) from brutal. Biologists study how we are alive and what makes us human, as distinct (or not) from other animals, once called brutes.

Regardless, the promise of applications to daily life often provides the most welcome justification to non-scientists for the pursuit of science. At a personal level, I am frequently asked how I apply my research, usually phrased as “What disease are you trying to cure?” Accepting the premise, I usually launch into an explanation of how basic science provides the foundation for applied science, which in turn has the conscious goal of treating disease to prolong healthy lives. My discourse culminates with the grandiose revelation that my work has been used, mostly by other people, to gain insights into pain syndromes, movement disorders and epilepsy. This assertion generally earns me an approving nod. Some impulse, however, makes me confess that my own research is not actively directed toward alleviating a particular illness. My interlocutor’s eyes narrow at what sounds like an admission that my contribution to the world is purely incidental. In that moment, I am as dismissed as the professor studying the 200 year old book.

**Figure 1:**
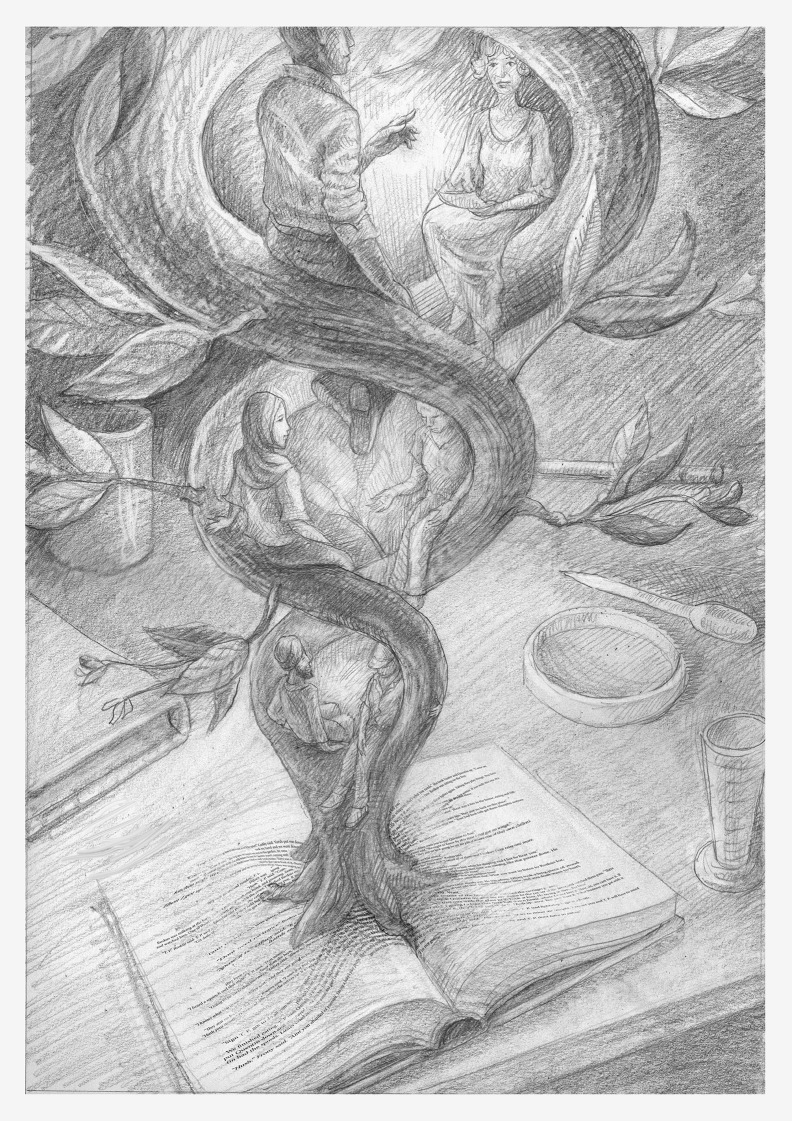
The assessments I heard were based on the work of minds and the achievements of hands.

Illustration: Ben Marder

My reply dissatisfies me, too; more exactly, I am exasperated at becoming ensnared yet again by the assumption that the sole responsibility of science is to fix tangible things – for the biological sciences, to produce medicinal drugs. As many scientists know, while the domain of science certainly encompasses immediate applications, it also extends beyond them, arcing into areas more broadly societal and humane.

Doing science shapes the experimentalist him-or-herself. When conducting experiments, you must subordinate yourself to physical reality; you must temper your expectations to conform to the sensitivities and proclivities of the subject under investigation – in biology, usually the cells or tissues or animals. You must acknowledge your errors, which are called out in no uncertain terms when experiments do not yield the anticipated outcomes. Even as you strive for dominion over a phenomenon, you are not always in command, as previously unknown variables sneak up and exert unpredicted effects. In other words, you don’t always get what you want. Instead, you must master and continually practice a form of self-control that, channeled properly, can subjugate any natural tendencies toward self-centeredness. True, those supreme moments of understanding, when a mystery seems solved or an answer attained, can bring out arrogance and egotism in some practitioners of science. Yet many other scientists – often less visible – incorporate the lessons of adversity more deeply, and they respond even to occasions of triumph with humility and awe at the workings of nature.

A year ago, I co-taught an undergraduate course with a colleague in the English Department. While exploring how literature and neuroscience each teach us to think about thinking, we read a series of novels that were, on average, just over 200 years old. From Ophelia’s depression in *Hamlet* to Benjy Compson’s mental disabilities in *The Sound and the Fury,* the plights and pathos of characters so vividly depicted by the authors of past centuries could be read as a call to treat the afflicted. It was almost with shame that I told my students that modern neuroscience still could not fully heal the people once referred to as madwomen and idiots. And yet, by explaining how the language has changed – by telling them how science has helped reject those terms along with the attitudes they fostered – I found myself presenting an aspect of science I had previously stopped short of articulating: Even where science has not yet cured, it has still helped us understand.

Owing to scientific inquiry, physical conditions formerly attributed to demons, devils, witches, judgments and unclean spirits can now be explained by genetics, nutrition, pathogens or physical damage. They have been liberated from the realm of retribution by supernatural beings and brought into the domain of action by compassionate humans beyond the saintly few. For people with epilepsy, cystic fibrosis, autism syndromes, intellectual disability and affective disorders, to name a few, the consequent improvement in their quality of life – and their ability to contribute to the lives of others – has been incalculable.

Although some purists may still interpret genetic anomalies as expressions of divine retribution or diabolical fury, for many others, scientific exploration has provided relief from the fear and cruelty that so often spring from unsubstantiated explanations of cause and effect. And with that accomplishment in mind, there is really only one disease that I see myself as actively trying to cure, and that is ignorance. It is the ailment, I think, that has caused more human suffering than all the neurological disorders put together, and it is the disease that all scientists – indeed, all educators – can help to cure.

Late last autumn, I attended a meeting of some 30,000 neuroscientists, one of the latest gatherings in the tradition of international scientific exchange begun well over 200 years ago. With the question of scientists’ responsibility to the larger community weighing on my mind, I looked around at the selection of humanity at the conference. Despite the sobriety of my reflections, I could not help feeling a small delight in seeing the nationalities, races, genders, generations and orientations all represented. Wherever I turned, evidence for a variety of religious and cultural backgrounds was on display – a purple turban, a head of green-and-turquoise-dyed hair, a brightly patterned hijab, an ultramarine baseball cap with red lettering. Lively discussion and spirited debates were taking place everywhere, but nobody seemed to care about the cloths or colors adorning each others’ heads. The assessments I heard were based on the work of minds and the achievements of hands: the quality of the voltage clamp, the resolution of the imaging tools, the selectivity of the antibodies and, most importantly, the integrity of the scientific logic.

There is really only one disease that I see myself as actively trying to cure, and that is ignorance

To me, it was quite beautiful; a living demonstration that, whatever the horrors of misunderstanding in the larger world engulfing us, for a tiny sliver of the globe’s population, the ideal of humanity united by education and exploration was not only possible but thriving. This is what it is like when it works, I kept thinking; here, too, is science in service to human health.

